# Social media for health promotion in diabetes: study protocol for a participatory public health intervention design

**DOI:** 10.1186/s12913-018-3178-7

**Published:** 2018-06-05

**Authors:** E. Gabarron, M. Bradway, L. Fernandez-Luque, T. Chomutare, A. H. Hansen, R. Wynn, E. Årsand

**Affiliations:** 1 Norwegian Centre for E-health research, University Hospital of North Norway, Sykehusvegen 23, 9019 Tromsø, Norway; 2 Department of Clinical Medicine, Faculty of Health Sciences, The Arctic University of Norway, 9019 Tromsø, Norway; 3 Qatar Computing Research Institute, Hamad Bin Khalifa University, Hamad Bin Khalifa Research Complex, Education City, Doha, Qatar; 4 Department of Community Medicine, University Hospital of North Norway, 9016 Tromsø, Norway; 5 Department of Community Medicine, Faculty of Health Sciences, The Arctic University of Norway, 9019 Tromsø, Norway; 6 Division of Mental Health and Addictions, University Hospital of North Norway, 9016 Tromsø, Norway

**Keywords:** Diabetes, Health promotion, Health education, Social media, Facebook, Twitter, Instagram, Community-based participatory research

## Abstract

**Background:**

Participatory health approaches are increasingly drawing attention among the scientific community, and could be used for health promotion programmes on diabetes through social media. The main aim of this project is to research how to best use social media to promote healthy lifestyles with and within the Norwegian population.

**Methods:**

The design of the health promotion intervention (HPI) will be participatory, and will involve both a panel of healthcare experts and social media users following the Norwegian Diabetes Association. The panel of experts will agree on the contents by following the Delphi method, and social media users will participate in the definition of the HPI by expressing their opinions through an *adhoc* online questionnaire. The agreed contents between both parties to be used in the HPI will be posted on three social media channels (Facebook, Twitter and Instagram) along 24 months. The 3 months before starting the HPI, and the 3 months after the HPI will be used as control data. The effect of the HPI will be assessed by comparing formats, frequency, and reactions to the published HPI messages, as well as comparing potential changes in five support-intended communication behaviours expressed on social media, and variations in sentiment analysis before vs during and after the HPI.

The HPI’s effect on social media users’ health-related lifestyles, online health behaviours, and satisfaction with the intervention will be assessed every 6 months through online questionnaires. A separate questionnaire will be used to assess the panel of experts’ satisfaction and perceptions of the benefits for health professionals of a HPI as this one.

**Discussion:**

The time constraints of today’s medical practice combined with the piling demand of chronic conditions such as diabetes make any additional request of extra time used by health care professionals a challenge. Social media channels provide efficient, ubiquitous and user-friendly platforms that can encourage participation, engagement and action necessary from both those who receive and provide care to make health promotion interventions successful.

## Background

The prevalence of diabetes is substantially increasing globally. In 2014 it was estimated that 422 million adults had diabetes, a number around four times higher then the 108 millions in 1980 [1]. This is a serious and chronic disease, that can lead to severe complications and premature death [1]. After the diagnosis has been confirmed, prevention actions are initiated that aim to reduce factors that can worsen the health of the affected and reduce the risk of premature death. These risk factors include tobacco use, an unhealthy diet, physical inactivity and excessive consumption of alcohol, etc. [1, 2]. The adequate education and counselling of patients with diabetes and their families has been emphasized by the World Health Organization [1]. These efforts have also been highlighted by the Norwegian Ministry of Health and Care Services as a key measure to promote patient commitment and self-treatment, and to reduce the development of complications [2]. However, medical practitioners may not always have the time to provide adequate and detailed answers to questions, or to offer health education or counselling to patients and their families during the infrequent and brief consultations they are allotted.

Social media channels have been proposed as effective educational tools through which to promote secondary prevention measures and behaviour change [3–10], and could also represent an effective platform for answering questions from patients and their relatives. Social media channels are powerful outlets for public health promotion due to their cost-effectiveness, precise evaluations of campaign success, and increased sustainability [11]. By the end of 2016, 2.34 billion users worldwide were using social media, and it is estimated that there will be 3 billion users by 2020 [12]. In high-connected countries such as Norway, social media are becoming increasingly important to seek out and share health information [13]. Evidence suggests that nowadays patients could be seeking and sharing health information on social media [13–19] as an additional resource to the consultations with their clinicians [20, 21]. However, although much of the health information available on social media seems to be of reasonably good quality [19], social media users are subject to risks associated with misleading or inadequate health information [19, 22].

Public health institutions, healthcare professionals, and other stakeholders could be more actively participative in these outlets, not only for answering diabetes patients’ questions, and correcting possible misinformation; but also taking advantage of the popularity of these channels to provide relevant health information for people with diabetes. In this sense, a participatory program in which the contents of the health promotion are agreed upon with people affected with diabetes has the potential to engage the target audience, and therefore to enhance patients’ wellbeing and satisfaction, and to improve health outcomes [23]. These participatory health approaches are increasingly drawing attention among the scientific community [24–27], and could be used for health promotion programmes on diabetes through social media.

## Methods/design

### Aim, design and participants

The main aim of this project is to research how to best use social media to promote healthy lifestyles with and within the Norwegian population. Secondary objectives of the project are:

- To analyze health behaviour on social media
- To analyze online discussions concerning diabetes
- To suggest systems and procedures to improve usage of social media for the dissemination of health information

The design of the health promotion intervention (HPI) will be participatory, and will involve both a panel of healthcare experts and social media users from the Norwegian Diabetes Association [28]. On one side, a panel of five healthcare professionals with an expertise in diabetes and/or patient health education will agree on the contents that will be used for the health promotion intervention. The panel of experts will agree on the contents by following the Delphi method [29, 30].

On the other side, all social media users from the Norwegian Diabetes Association will be invited to actively participate in the definition of the HPI by expressing their opinions through an *adhoc* online questionnaire regarding contents, frequency and format. The questions included in the questionnaire will be agreed between researchers, healthcare professionals and personnel from the Norwegian Diabetes Association. The online questionnaire will be distributed using LimeSurvey, an open source online survey tool [31]. Links to the online questionnaire will be posted on the three social media channels (Facebook, Twitter and Instagram). The online questionnaire will not track any information that can identify or trace users. The questionnaire will include questions regarding topics of interests, preferred frequency of the messages, preferred format of the information, and preferred social media channels.

The agreed contents to be used in the health promotion intervention (between the panel of experts and social media users of diabetes groups) will be posted on the social media channels. These messages will draw on the Laugh Model [11], a framework that proposes using social media to change behaviour [11]. Because social media are attractive platforms through which the intervention can be presented, users are more likely to engage in them.

### Intervention

The HPI is expected to start at the end of 2017, and last until the end of 2019. The HPI will last 24 months in total, and will be carried out through the Norwegian Diabetes Association, Diabetesforbundet [28] and other relevant organizations’ social media channels, with the aim of reaching over all their social media users (over 35,000 by October 2017) [28]. During the first 12 months of the intervention (Phase I), health messages promoting secondary prevention measures and behaviour change for diabetes will be disseminated through the social media channels. In the following 12 months (Phase II), in addition to the health messages, the HPI will include an “ask the experts” service, whereby people with diabetes will be able to send their questions.

### Control

The 3 months before starting the HPI, and the 3 months after the HPI will be used as control data. The formats, frequency, and reactions to the published HPI messages on the 3 social media channels (Facebook, Twitter, and Instagram) will be tracked during the 3 months prior and 3 months after the HPI.

Patterns of users’ online discussions and behaviours related to diabetes will also be actively monitored for 30 months: 3 months before starting the HPI, during Phase I, during Phase II, and 3 months after the HPI. All of the contents tracked on social media will be analysed according to the Social Support Behaviour Code [32] regarding the five support-intended communication behaviours: 1) information support, i.e. providing information or advice; 2) esteem support, i.e. communicating respect and confidence in abilities; 3) network support, i.e. communicating belonging to a group of persons with similar concerns or experiences; 4) emotional support, i.e. communicating love, concern, or empathy; and 5) tangible assistance, i.e. providing, or offering to provide, goods or services.

Additionally, content sentiment analysis will be carried out during the whole study (3 months before starting the HPI, during Phase I, Phase II, and 3 months after the HPI). Sentiment analysis will involve automatic classification of social media’ comments to the HPI messages into positive, neutral and negative feelings [33]. Sentiment analysis will be used as outcome to assess the impact of the health promotion intervention.

### Outcomes

#### Primary outcome

The primary outcome is the effect of the participatory social media health promotion intervention on the reported health-related lifestyles and online health behaviours of people living with diabetes.

Users of diabetes social media groups will be surveyed through an *adhoc* questionnaire in order to assess the HPI’s effect on their health-related lifestyles, and also on their online health behaviours. The questionnaire will be distributed before starting the HPI, and every 6 months during the 24 months of the HPI, i.e. during Phase I, during Phase II, and at the end of the health promotion intervention.

A questionnaire will be also distributed to the health professionals involved in the project, to the panel of experts, to assess their satisfaction and their perceptions of the benefits for health professionals of a HPI such as this one.

#### Secondary outcomes

Secondary outcomes will be assessed through monitored contents on the study participants’ social media channels. These are: 1) identification of positive measures regarding the five support-intended communication behaviours, these behaviours will be identified and assessed according to the Social Support Behaviour Code [32]; 2) an increase in positive mood of diabetes patients during the health promotion intervention, which will be assessed with automatic classification‘sentiment analyses tools; and 3) an increase in perceived quality of health information after the health promotion intervention, which will be compared with the perceived quality of health information assessed before the intervention.

Analysis of the results derived from the users’ involvement, the HPI, the monitoring of online health information, and the monitoring of health behaviour will be used to suggest systems and procedures for the Norwegian Diabetes Association and other stakeholders to improve their usage of social media for future health promotion interventions.

### Statistical methods

The opinions of social media users and healthcare professionals revealed by the questionnaires will be analysed using descriptive statistics. Results will be expressed in form of frequencies and percentages for each categorical variables and mean, standard deviation (SD) and 95% confidence interval (95% CI) for continuous variables. T-Tests, ANOVA, correlation and Chi-Square analyses will be performed as well. Descriptive statistics will also be used to summarize positive and negative mood in sentiment analysis; and also the 5 types of social support found on social media as reactions to the participatory health promotion intervention: 1-information support; 2-esteem support; 3-network support; 4-emotional support, and 5-tangible assistance, according to the Social Support Behaviour Code [32].

Quantitative data analysis will be performed with the latest version of the SPSS statistical package. For the sentiment analysis we will use any of the available tools in Norwegian language (e.g. Polyglot, Lexalytics, or similar).

## Discussion

Participatory health intervention approaches and collaborative research involving researchers and community representatives are increasingly drawing attention among the scientific community [24–27], and could be used for health promotion programmes on diabetes through social media.

Within the Norwegian health services, there is little systematic use of social media to promote healthy lifestyles or offer advice to its users. While the reason for this is unclear, one might speculate that the traditional health services are slow in adopting new means of communication with their users [34]. However, the time constraints of today’s medical practice combined with the piling demands caused by chronic conditions such as diabetes make any additional request on the time of health care professionals a challenge. Social media channels provide efficient, ubiquitous and user-friendly platforms that can encourage participation, engagement and action necessary from both those who receive and provide care, to make health promotion interventions successful.

In the present project, there will be collaboration between patient users, patient user organizations, and health professionals/researchers. The HPI will be developed jointly by the participants and will rely heavily on feedback from real patient users. The project is unique and innovative in the Norwegian setting and may provide important insights that can be used for other health promotion interventions drawing on social media and heavy user involvement in Norway.

This research project will investigate the use of a participatory approach to promote healthy lifestyles on social media among Norwegians with diabetes. The project will contribute to improving quality of care and quality of life while reducing social inequalities in health, since it is based on media that are available and accessible for all. The use of a participatory approach can potentially increase diabetes patients’ engagement and satisfaction with the health promotion intervention, and therefore help people attain healthier lifestyles and the intervention can also provide benefits for participating health professionals and the health service.

## Abbreviations

HPI: Health promotion intervention
